# Regulation of *MYC* Expression and Differential JQ1 Sensitivity in Cancer Cells

**DOI:** 10.1371/journal.pone.0087003

**Published:** 2014-01-23

**Authors:** Trent Fowler, Payel Ghatak, David H. Price, Ronald Conaway, Joan Conaway, Cheng-Ming Chiang, James E. Bradner, Ali Shilatifard, Ananda L. Roy

**Affiliations:** 1 Department of Developmental, Molecular and Chemical Biology, Tufts University School of Medicine, Boston, Massachusetts, United States of America; 2 Biochemistry Department, University of Iowa, Iowa City, Iowa, United States of America; 3 Stowers Institute for Medical Research, Kansas City, Missouri, United States of America; 4 Simmons Comprehensive Cancer Center, University of Texas Southwestern Medical Center, Dallas, Texas, United States of America; 5 Dana Farber Cancer Institute, Harvard Medical School, Boston, Massachusetts, United States of America; 6 Program in Genetics, Sackler School of Biomedical Science, Tufts University School of Medicine, Boston, Massachusetts, United States of America; 7 Program in Immunology, Sackler School of Biomedical Science, Tufts University School of Medicine, Boston, Massachusetts, United States of America; Wayne State University, United States of America

## Abstract

High level MYC expression is associated with almost all human cancers. JQ1, a chemical compound that inhibits MYC expression is therapeutically effective in preclinical animal models in midline carcinoma, and Burkitt’s lymphoma (BL). Here we show that JQ1 does not inhibit MYC expression to a similar extent in all tumor cells. The BL cells showed a ∼90% decrease in MYC transcription upon treatment with JQ1, however, no corresponding reduction was seen in several non-BL cells. Molecularly, these differences appear due to requirements of Brd4, the most active version of the Positive Transcription Elongation Factor B (P-TEFb) within the Super Elongation Complex (SEC), and transcription factors such as Gdown1, and MED26 and also other unknown cell specific factors. Our study demonstrates that the regulation of high levels of MYC expression in different cancer cells is driven by unique regulatory mechanisms and that such exclusive regulatory signatures in each cancer cells could be employed for targeted therapeutics.

## Introduction

Lymphomas are broadly classified into two categories: Hodgkin’s and non-Hodgkin’s lymphoma (NHL) [1]. The two most common forms of aggressive NHL are diffuse large B cell lymphoma (DLBCL) and Burkitt’s lymphoma (BL) [1], [2], [3]. Translocation of the proto-oncogene *MYC* into one of the immunoglobulin gene loci [*IG-MYC* translocation; mostly of the t(8;14)q24;q32 type] resulting in aberrant *MYC* expression is regarded as the dominant genetic event in the genesis of BL and about 10% of DLBCL [4]. Besides translocation, *MYC* can undergo oncogenic deregulation via high-level gene amplification as well as mutations in cis-regulatory elements in several cancer types (e.g., myeloma, colon carcinoma and neuroblastoma) [5], [6]. Moreover, while most BLs have deregulated c-*MYC* (henceforth referred to as *MYC*) expression as a consequence of an *IG-MYC* translocation, the majority of non-BLs do not carry *IG-MYC* translocations, but other genetic abnormalities leading to deregulated *MYC* expression. Though high expression is restricted to BLs, MYC target expression varies on a lower level across non-BL and intermediate lymphomas and constitutes a negative prognostic marker in these lymphomas.

MYC dimerizes with MAX to bind its target sequence (E-box) and regulate gene expression. MYC not only activates transcription but also represses target gene expression either via direct biding (with Miz-1 transcription factor) or via regulation of micro-RNAs (miRs) [7]. However, the function of MYC is complicated as two recent studies show that MYC does not have a specific transcriptional signature but serves to amplify the output of existing transcriptional programs in a given cell rather than executing its own transcriptional program [8], [9].

*MYC* belongs to a class of genes called Primary Response Genes (PRGs) many of which harbor paused Pol II at the proximal promoter area that upon activation can quickly switch to an elongating Pol II and functional transcription [10]. Signal dependent stimulation results in enhanced acetylation at histone 3 lysine 14 (H3K14Ac) and either of two H4 lysine pairs, H4K5/12 or H4K8/16, an event which appears crucial for the binding of bromodomain (BRD) proteins that recruit transcription factors necessary for transcription [11], [12]. Recruitment of positive transcription elongation factor b (P-TEFb), and possibly the general initiation cofactor Mediator, plays an important role in Brd4-regulated transcription of many genes including *MYC*. Indeed, after recruitment by Brd4, P-TEFb phosphorylates the elongation factors DSIF (Spt4 and Spt5), negative elongation factor (NELF), and Pol II resulting in release of paused Pol II and subsequent elongation of transcription [10], [13] and recently reviewed in [14]. Collectively, these and other results suggest that elongation of transcription is a critical regulatory point in orchestrating *MYC* regulation [10].

As many MYC-driven processes are required for homeostasis and growth, therapeutic strategies geared towards modulation of *MYC* expression rather than outright suppression seem attractive. The Mitsubishi Tanabe Pharma Corporation first described thienodiazepine analogs that potently inhibit chromatin binding of bromodomains related to the BET family [15]. Subsequently, a closely related small molecule inhibitor, JQ1, has been found to be therapeutically effective in pre-clinical animal models [16], [17], [18]. These and related small molecules competitively occupy the acetyl-binding pockets of BET bromodomains, resulting in release of BET proteins (in particular Brd4) from chromatin [19]. Despite these promising demonstrations, JQ1 does not inhibit *MYC* expression to a similar extent in all tumor cells [19]. Thus, it is important to understand why JQ1 is effective in only certain *MYC*-dependent cancers, which may ultimately determine clinical efficacy of this compound in patients.

We observed that although JQ1 treatment decreased Brd4 occupancy to a similar degree in the cell types tested, the ability of JQ1 to reduce *MYC* transcription between cells differed. In JQ1-sensitive cells, inhibition occurred at the level of nascent transcription affecting Pol II, “paused” Pol II-Ser5-P, “elongating” Pol II-Ser2-P, and P-TEFb occupancy. Furthermore, there is a difference in the occupancy of members of the Super Elongation Complex (SEC), and factors associated with RNA Polymerase II, at *MYC* promoter regions in the two cell types. Collectively, our data indicate high levels of *MYC* are maintained in different cancer cells via distinct mechanisms at the level of transcription elongation with different compliments of transcription factors and are therefore subjected to different JQ1 sensitivity.

## Materials and Methods

### Cell Culture

The mature mouse B cell lymphoma BAL17 [20], human BL lines Akata [21], Raji [22], and Ramos [23], and human epithelial line HeLa-S3 [24] were cultured in RPMI media with HEPES (Invitrogen) supplemented with 100 U/ml Penicillin, 100 µg/ml Streptomycin, 1 mM Sodium Pyruvate, 0.5 mM 2-Mercaptoethanol solution (Invitrogen) and 10% Fetal Calf Sera (Atlanta Biologicals). RNA based assays used 1–2×10^6^ cells and 2×10^7^ cells were used for Chromatin Immunoprecipitation (ChIP). JQ1, solubilized in DMSO, was diluted into media at 1 µM and incubated with cells for 2 hours at 37°C. No effects on transcription were observed with DMSO controls (data not shown). Experiments shown in Figure S1 (File S1) involving BCR stimulation consisted of a 2 hr pre-treatment with 1 µM JQ1 followed by 30 minutes of exposure to antibody fragments specific for either human or mouse BCR (Jackson Immunoresearch).

### RNA-Real Time PCR Analysis

Performed as previously described [25]. Ct values were averaged and the signal reported as the ratio of target over ActB mRNA via linear equations specific to each primer pair. Myc primers are listed in S3 (File S1) unless shown below.

### mRNA Primers-mouse

Myc Primers not listed in Figure S3 (File S1)

In1/Ex1 5′-AGAGCTCCTCGAGCTGTTTG

5′-CGTCTACATTCAAGACGCAGA

ActB  5′-AGGCATGGAGTCCTGTGGTATC

5′-AGCCACAGGTCCTAAGGCCAG.

Fos  5′-GGATTTGACTGGAGGTCTG

5′- TGGGCTCAGGGTCGTTGA

### mRNA Primers Human

Myc Primers not listed in Figure S3 (File S1)

In1/Ex2 5′-GCACCAAGACCCCTTTAACTC

     5′-TCCTGTTGGTGAAGCTAACGEx2/In2 5′-AGCGACTCTGGTAAGCGAAG

5′-GTGGCCCGTTAAATAAGCTG

ActB    5′-CTCTTCCAGCCTTCCTTCCT

5′-AGCACTGTGTTGGCGTACAG

Fos    5′-CTCCGGTGGTCACCTGTACT

5′-GTCAGAGGAAGGCTCATTGC

### Western Blotting

Nuclear extracts from 2×10^6^ cells were subjected to 10% (Myc) or 6% (Brd4) SDS-PAGE followed by Western blotting. Primary antibodies (rabbit anti-C-terminal Brd4-C IgG and rabbit anti-Brd4-S484/488-phos) [26], [27], rabbit anti-CREB IgG (Cell signaling) and secondary (goat anti-rabbit horseradish peroxidase-linked IgG, Invitrogen) antibodies at 1∶1500. Blots were visualized with the Novex ECL chemo-luminescent Substrate Regent Kit (Invitrogen) and densitometry performed with the ChemiDoc MP Imaging System (Biorad).

### Strand-specific Detection

380 ng of RNA with 1 µM final primer concentration were employed for each RT reaction using Invitrogen’s Cloned AMV First Strand synthesis kit. Strand-specific primers; for (+) strand the reverse primers corresponding to the TSS area (+17 for mouse and +11 for human) and for (−) strand forward primers corresponding to the end of the gene (+3948 for mouse and +4791 for human, sequences in Figure S3, in File S1). Standard real-time PCR amplification of the was employed with the indicated primers and their companions, shown in Figure S2 (File S1), and the resulting Ct values were converted to relative ng and normalized to the starting concentration of chromosomal RNA.

### Chromatin Immunoprecipitation (ChIP)

A standard ChIP assay was performed and has been previously described [25]. PCR primers and scheme are shown in Figure S3, in File S1. Ct values were averaged and the signal represented as % of input DNA via linear equations specific to each primer pair.

### ChIP Antibodies

Rabbit polyclonal anti-mouse RNA Pol II (N-20, sc-899, Santa Cruz), RNA Pol II-Ser5P mouse monoclonal (sc-47701, Santa Cruz), RNA Pol II-Ser2P H5 mouse ascites (Covance), H3K36me3 Rabbit polyclonal (ab9050, Abcam), anti-Cyclin T rabbit polyclonal (H-245, sc-10750, Santa Cruz), rabbit anti-C-terminal BRD4 IgG [27], MED26 mouse anti-MED26/CRSP7 (Ab50619, Abcam), Gdown1 sheep anti-Gdown1 IgG [28], anti-AFF4 antibody [29] and anti-ELL2 antibody [29].

### Sequencing Analysis

Figure S2 (File S1) shows sequencing data derived from University of California Santa Cruz Genome Browser (UCSC GB) tracks “wgEncodeUtaChIPseqBaseOverlapSignalHelas3Pol2” (HeLa S3) and GEO data set GSM920942 (Raji), mapped to human genome build hg18. The University of California Santa Cruz Genome Web Browser setting normalized to the highest peak at the TSS.

## Results

### Differential JQ1 Sensitivity

**Figure 1:**
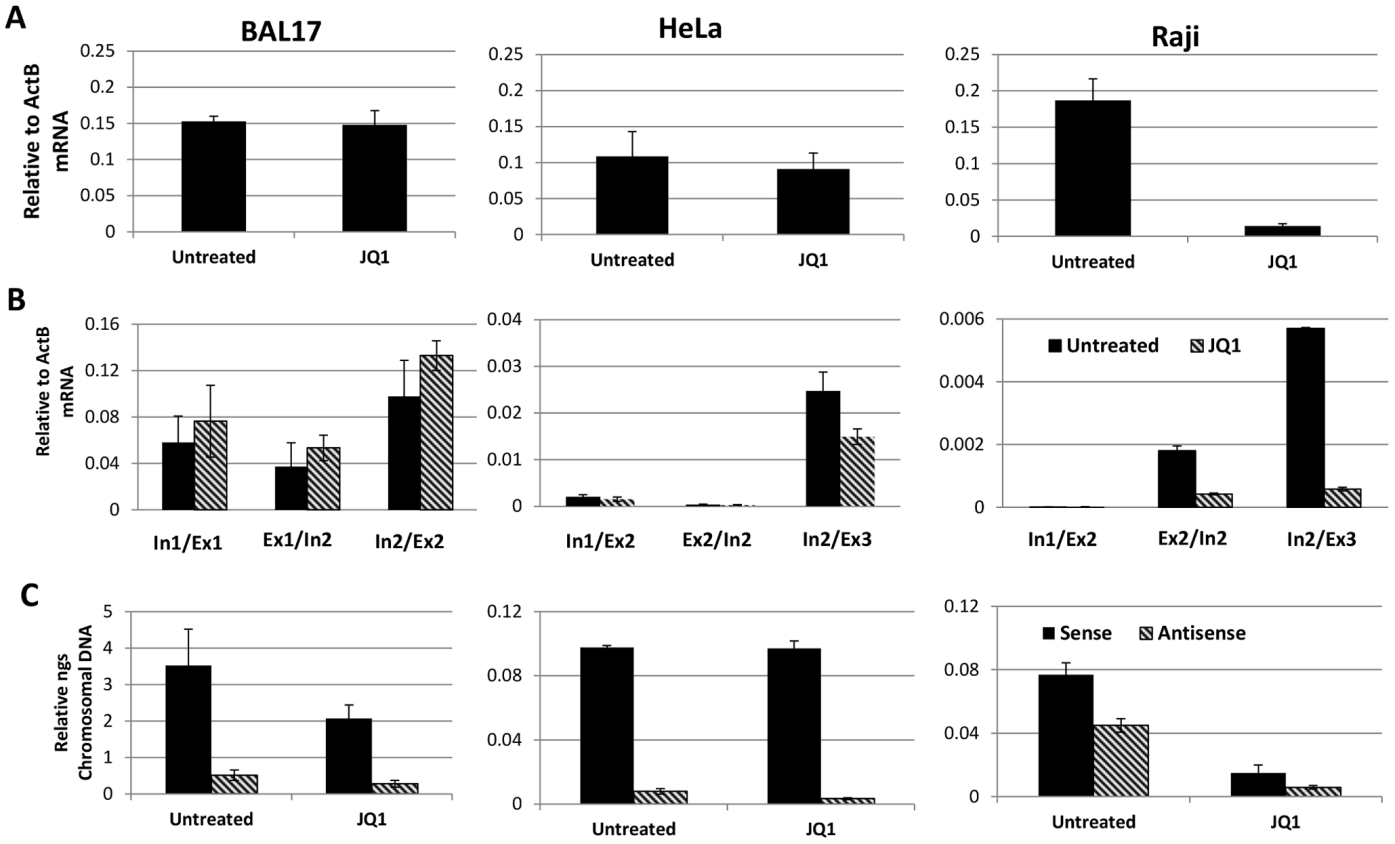
Effects of JQ1 on *MYC* expression. BAL17 (Murine B cell), Human HeLa, and Human BL Raji cells and either untreated or treated with 1 µM of JQ1 for 2 hours. (A) mRNA analysis was performed in triplicate, reported relative to ActB mRNA expression and is shown as the average and standard deviation of three experiments. (B) Detection of primary transcription by PCR amplification using primers across internal exon/intron borders of *MYC* was performed three times and reported relative to ActB mRNA expression. (C) Strand-specific transcription was detected by strand specific reverse transcription amplification as detailed in Material and Methods followed by standard PCR methods. These experiments were performed twice.

We performed dose- and time-dependent testing of Brd4 inhibition with JQ1 and noted the effect on *MYC* transcription in a variety of cells including HeLa-S3 cells, whose MYC expression was reported [19] to be resistant to JQ1 treatment. Shown in Fig. 1A, tested cell lines express *MYC* at high levels, comparable to levels detected at the peak of primary naïve resting B cell induction (data not shown). Steady state mRNA levels of *MYC* in a non-BL murine B cell lymphoma line (BAL17) that over expresses *MYC* at levels similar to the BL cells and HeLa cells showed no significant sensitivity to JQ1 (Fig. 1A). Both HeLa and BAL17 cells continued this resistance at concentrations up to 5 µM (data not shown), while *MYC* RNA in BL cells was uniformly decreased by ∼90% when treated with 1 µM of JQ1 for a period of two hours (Fig. 1A). BL cells are reported to express 2- to 5-fold more *MYC*-specific RNA than B-cell lines without a translocation [30]. Although Western blotting d indicate the Raji BL line expresses more MYC protein, consistent with the mRNA data (Fig. 1A), JQ1 treatment decreased MYC protein expression in Raji but not HeLa and BAL17 (data not shown). Together these results demonstrated that JQ1 resistance occurred in both non-lymphoma and lymphoma cell lines representing both murine and human species.

Because mRNA production may not necessarily correlate with primary transcript production [25], [31], we tested the effect of JQ1 on primary transcription. Analysis of primary transcript, performed with primers against the exon/intron borders of *MYC* (schematically represented in Supplemental Fig. 3 and/or listed in Materials and Methods), showed that primary transcription sensitivity to JQ1 was parallel to that of mRNA (Fig. 1B). Thus, long, and presumably full-length, primary transcripts were either resistant (BAL17 or HeLa) or sensitive (Raji) to JQ1. Therefore, the difference in JQ1 responses correlated with primary transcription and did not occur at the level of post-transcriptional modification, such as splicing and mRNA or protein stability.

Divergent transcription is common of many promoters in organisms and plays an important role in gene regulation [32]. Indeed, antisense transcription from the *MYC* locus has been observed in several cell lines [33]. Because we only measured total steady state RNA (Fig. 1A), whether differential JQ1 sensitivity reflects differences in possible transcription originating from the 3′ end was tested. Although antisense transcription in both BAL17 and HeLa cells was small in comparison to sense transcription, it was largely unaffected by JQ1 (Fig. 1C, left and middle panels). The wide difference in levels of sense and antisense transcripts observed amongst different cell lines is currently unexplained but might reflect a combination of transcriptional and post-transcriptional effects. Regardless, like sense transcription, antisense transcription originating from the *MYC* locus in Raji cells was inhibited by JQ1 (Fig. 1C, right panel). Therefore, although more antisense transcription was noted in Raji cells, the difference in JQ1 sensitivity was not likely due to differences in antisense transcripts.

### JQ1 Leads to Decreased Brd4 Occupancy in Resistant and Sensitive Cells

**Figure 2:**
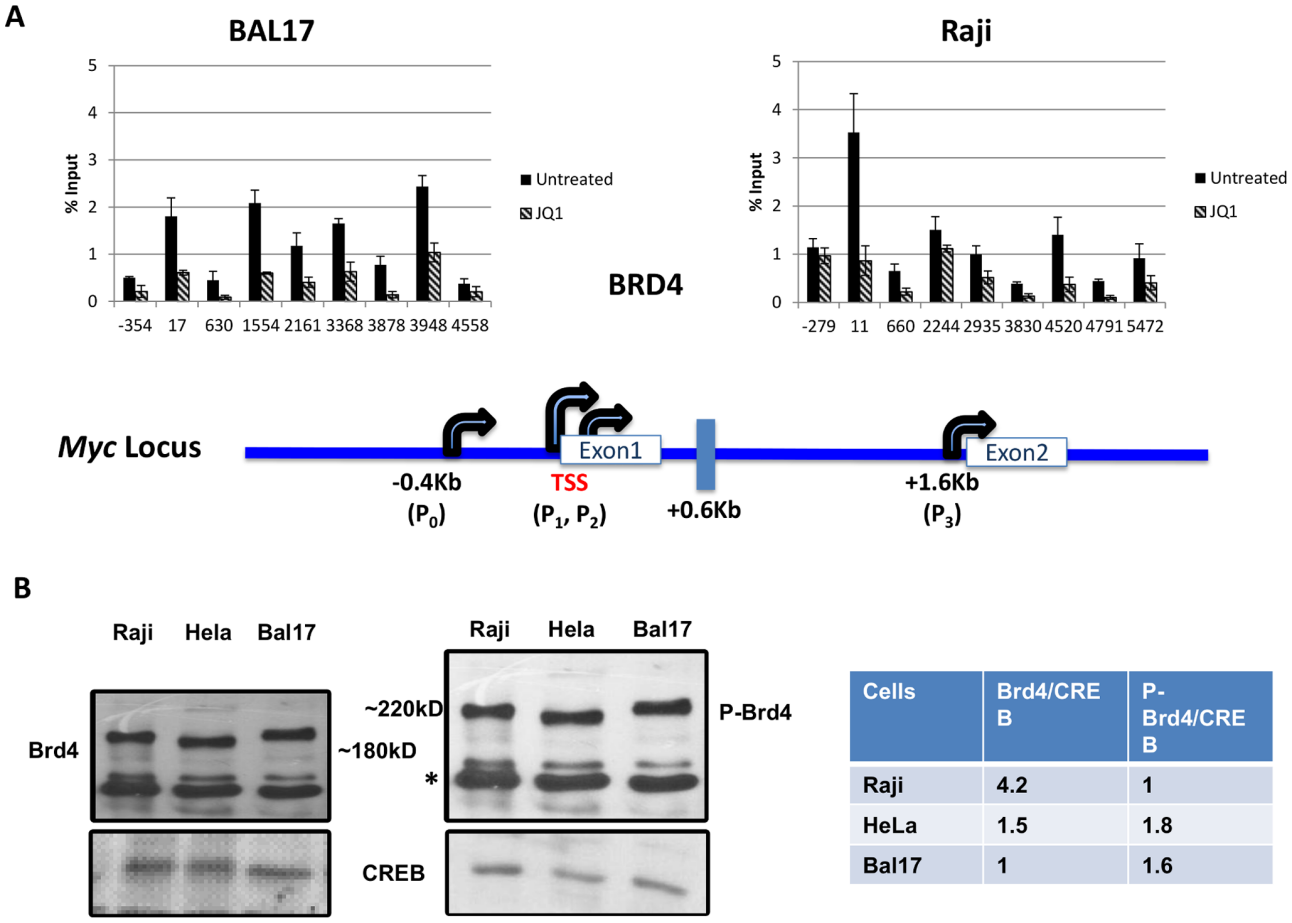
Brd4 occupancy and expression in different cells. Cells were either untreated or treated with 1 µM of JQ1 for 2 hours. (A) Chromatin Immunoprecipitation (ChIP) across *MYC* with anti-C-terminal Brd4 antibody. Each experiment was performed twice, analyzed in triplicate via real-time PCR and reported as the mean and standard deviation of the two experiments. A representation of the promoter area of *MYC* is provided for orientation. (B) Western blotting to detect (far left) Brd4 (∼180 KD) and (middle) Brd4-S484/488-phos (P-Brd4, ∼220 KD) was performed three times. A non-specific band detected with phopsho-Brd4 antibody is denoted with an asterisk. Typical results are shown with densitometry analysis relative to CREB expression, which is used as a normalization control (far right).

We observed that Brd4 recruitment was reduced by JQ1 in both cell types–therefore, the difference in JQ1 sensitivity between different cells was not due to permeability. We also observed that Brd4 recruitment to the transcription start site (TSS) was roughly 2.5 fold more in Raji (+11) cells compared to BAL17 (+17) (Fig. 2A). HeLa exhibited Brd4 recruitment and JQ1 sensitivity similar to BAL17 (data not shown). Moreover, a significant amount of Brd4 occupancy was noted across the body of *MYC* in all the cells tested, though there was a higher level of coding region occupancy in BAL17 cells. Though commonly associated with 5′-end enhancers and promoters, Brd4 has been shown to occupy the coding region of PRGs such as c-*fos* and *MYC* [26].

Promoter recruitment of Brd4 requires phosphorylation at S484 and S488 and deletion of this region results in decreased CycT and Pol II occupancy and transcription [26]. Consistent with the ChIP assay, Western blotting showed a higher level of total Brd4 in Raji nuclear extracts compared to HeLa and BAL17, although Brd4-S484P/S488P in Raji cells was slightly less compared to BAL17 and HeLa (Fig. 2B). Interestingly, the P-BRD4 band in HeLa cells migrated faster than either the Raji or BAL17 P-Brd4 band, perhaps reflecting a slight difference in the extent or site of phosphorylation. However, the difference in phosphorylated Brd4 (the active form) between different cells might not explain the differences in JQ1 sensitivity.

**Figure 3:**
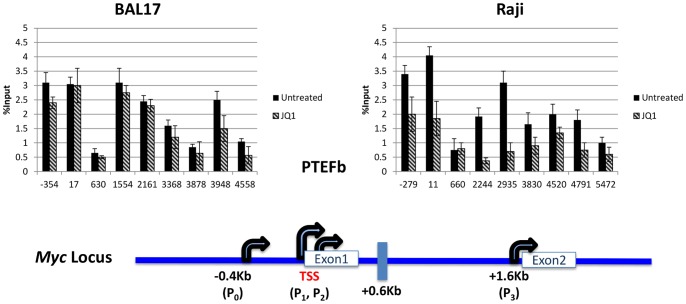
Effects of JQ1 on P-TEFb occupancy. BAL17 and Raji cells were either untreated or treated with 1 µM of JQ1 for 2 hours. Recruitment of P-TEFb was detected by ChIP assays. Each experiment was analyzed in triplicate via real-time PCR, performed twice, and is reported as the mean and standard deviation of the two experiments.

Brd4 interacts with factors that either recruit Pol II to the site of transcription or drive the transcriptional complex from pausing to the elongation mode (reviewed in [10]). Additionally, a reduction of functional Brd4 resulting in greatly reduced Pol II occupancy was shown to involve a loss of P-TEFb [26]. Although the level and pattern of P-TEFb occupancy in both untreated JQ1 sensitive and resistant cell types were similar, JQ1 treatment/Brd4 inhibition decreased P-TEFb occupancy only in Raji cells (Fig. 3). These results indicated levels of BRD4 dependence for maintaining P-TEFb occupancy vary in different cell lines (Fig. 3).

### RNA Pol II Recruitment

**Figure 4:**
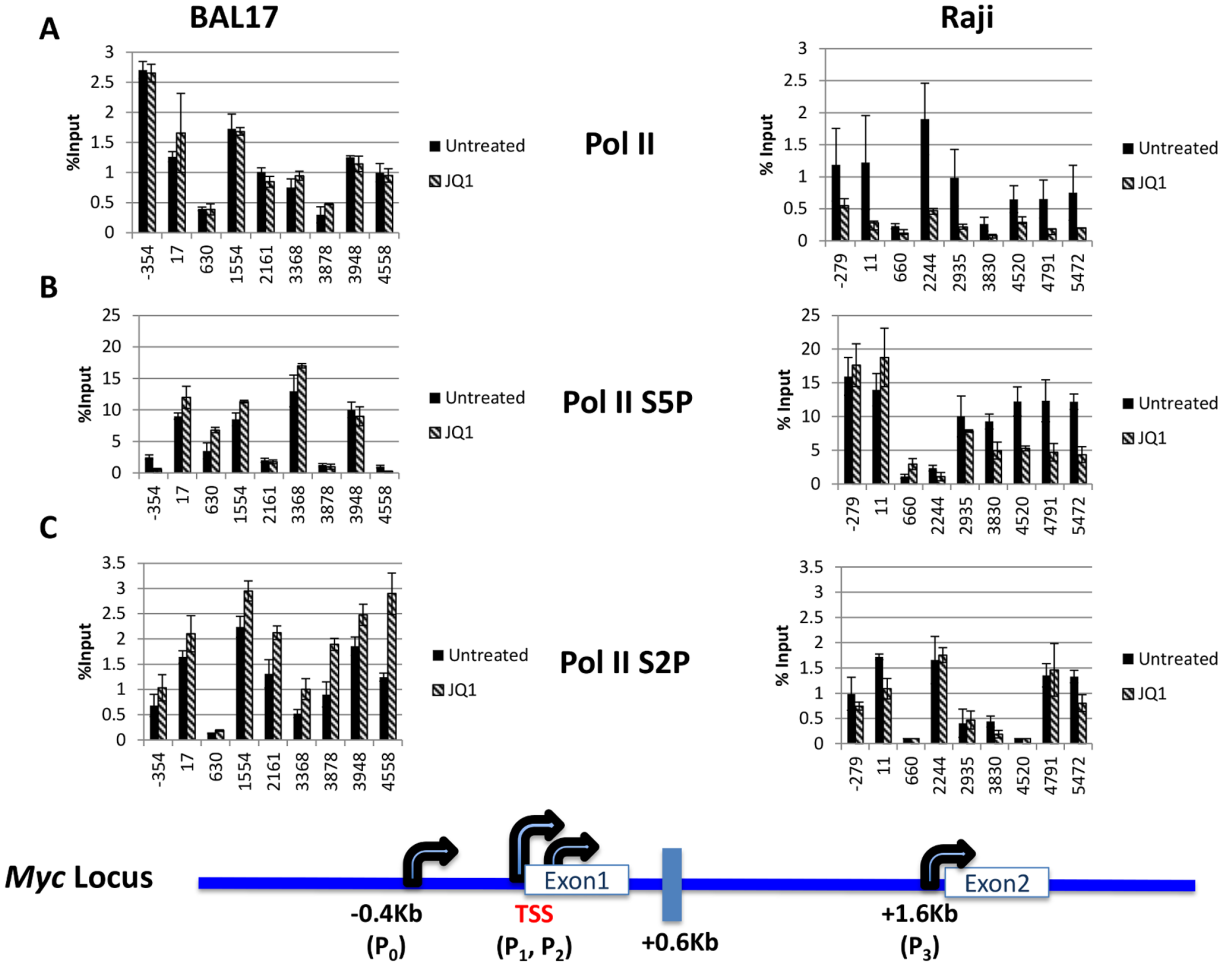
RNA Polymerase II occupancy in the absence and presence of JQ1. Cells were either untreated or treated with 1 µM of JQ1 for 2 hours. (A) Pol II was detected by antibody against the N-terminal of Pol II (B) Pol II Serine 5-P (C) Pol II Serine 2-P. Chromatin ChIP assays were performed in duplicate. Each experiment was analyzed in triplicate via real-time PCR, performed twice, and is reported as the mean and standard deviation of the two experiments.

We next determined if the difference in JQ1 selectivity was due to a difference in general transcription apparatus recruitment, represented by RNA Polymerase II (Pol II). While total Pol II was greatly decreased across the body of *MYC* in Raji cells upon JQ1 treatment, it was not altered in the JQ1 resistant BAL17 line (Fig. 4A). Both cell types showed Pol II occupancy at the P_0_ promoter, which has been shown to correlate with a very high level of transcription [34]. Many actively transcribed genes (including *MYC*) exhibit paused Pol II at their proximal promoters [35]. Consistent with this notion, we observed a high level of promoter-associated Pol II in both cell types, although two-fold more Pol II was noted at the proximal promoter (roughly corresponding to the P_0_ promoter region) in BAL17 (−354) compared to Raji (−279). Interestingly, as noted with Brd4 and P-TEFb (Figs. 3 and 4), total Pol II was reduced at a site immediately downstream (+0.6 kb) of the TSS and sharply increased afterward (Fig. 4A).

Because phosphorylation of the carboxy-terminal domain (CTD) of RNA Pol II is associated with regulation of transcription initiation and elongation at many promoters [36], [37], we analyzed these events in the two cell types in the absence and presence of JQ1. While RNA Pol II phospho-Ser5 (Pol II S5P) levels in BAL17 cells remained stable after JQ1 treatment, Pol II S5P levels in Raji cells were sensitive to JQ1 (Fig. 4B). However, surprisingly, Pol II S5P levels were refractory at P_0_ and TSS sites (Fig. 4B, right panel) but sensitive in the latter half of the gene with a small change starting downstream of the *MYC* P3 promoter (+2244) and becoming increasingly sensitive further down the body of the gene. Whether the substantial amount of Pol II-Ser5P 3′ occupancy shown here is directly due to 3′ Brd4 occupancy or an overabundance of transcription complexes “backing up” resulting in reduced elongation and subsequent pausing at the 3′ terminus is unknown.

While Ser5 phosphorylation is associated with a competent but paused Pol II, Ser2 phosphorylation of the Pol II CTD (Pol II S2P) represents elongating Pol II [37]. In the absence of JQ1, Pol II S2P occupancy in both cell types occurred across the body of the gene with peaks at the promoter regions, and the 3′ end. Surprisingly, while promoter- and TSS-associated Pol II S2P levels in Raji cells were sensitive to JQ1, downstream regions past P_3_ exhibited no JQ1 sensitivity until past the 3′ UTR (+5472) (Fig. 4C), suggesting that the majority of fully elongating RNA polymerase II in these JQ1 sensitive cells was refractory to JQ1. We also observed a JQ1-dependent increase in Pol II-Ser2P in the BAL17 line and while this increase was slight at the promoter and TSS regions, it was very clear at the 3′ terminus.

### Differences in H3K36me3

**Figure 5:**
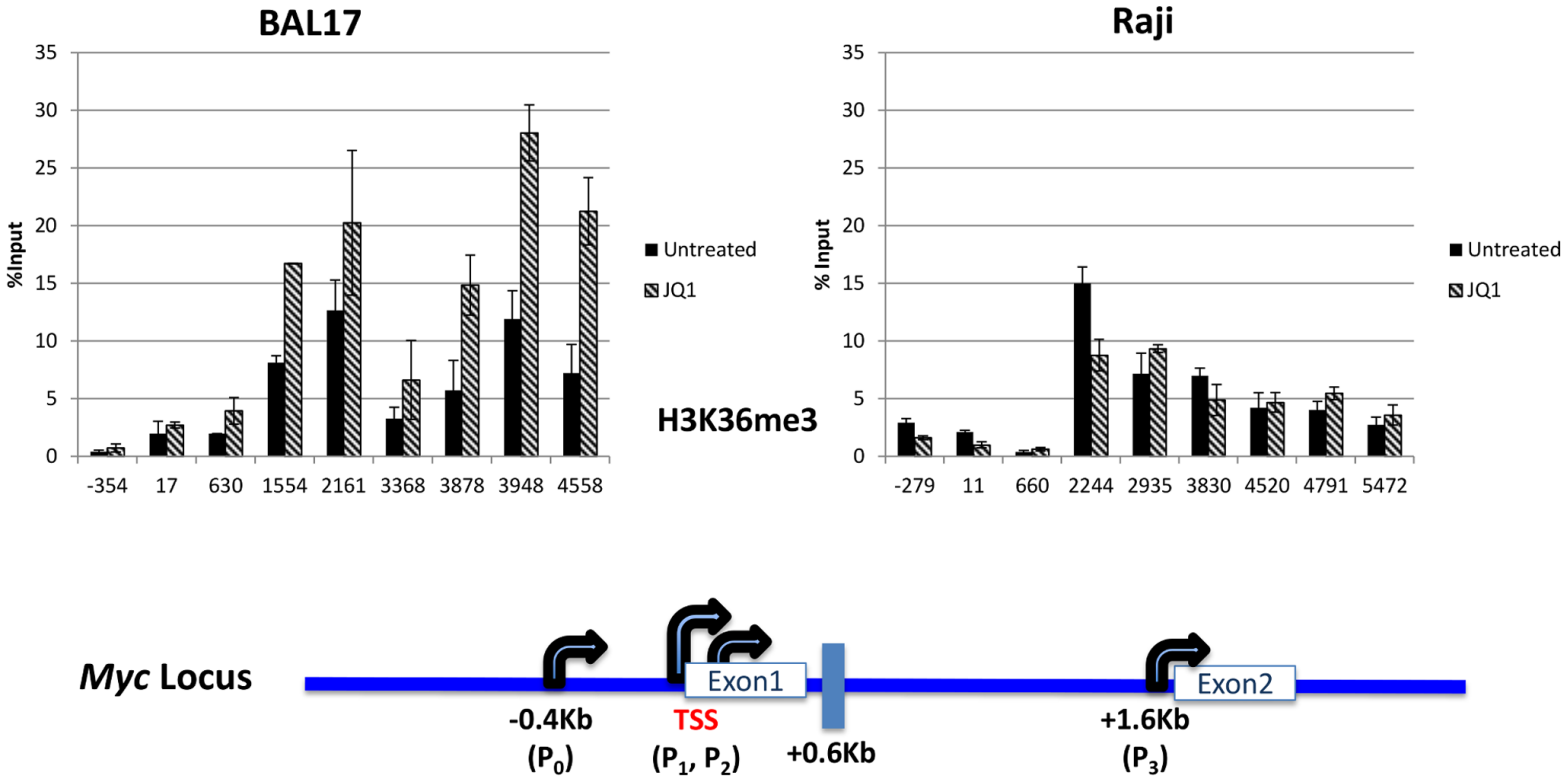
Effects of JQ1 on H3K36me3 status. Cells were either untreated or treated with 1 µM of JQ1 for 2 hours. ChIP assays were performed in duplicate. Each experiment was analyzed in triplicate via real-time PCR, performed twice, and is reported as the mean and standard deviation of the two experiments.

One possible way to delineate between upstream and downstream effects on Pol II-Ser2 occupancy is to determine the pattern of tri-methylation of the histone 3 residue lysine 36, H3K36me3, which is indicative of a recently passing elongating transcription complex [37]. In BAL17 cells, JQ1 treatment enhanced the H3K36me3 signals across the gene (Fig. 5). But in Raji cells, JQ1 treatment decreased H3K36me3 from P_0_ through TSS until the P_3_ region. However, beyond P3, the H3K36me3 became refractory to JQ1 (Fig. 5) as observed with Pol II S2P (Fig. 4C), suggesting that once RNA Pol II was in elongation mode, it was insensitive to JQ1. Together, our data showed that *MYC* harbored transcription complexes with variable Brd4 dependency in different cell types and that Brd4 inhibition resulted in a differential response at the level of transition from pausing to elongation.

### Accessory Transcription Elongation Factors

**Figure 6:**
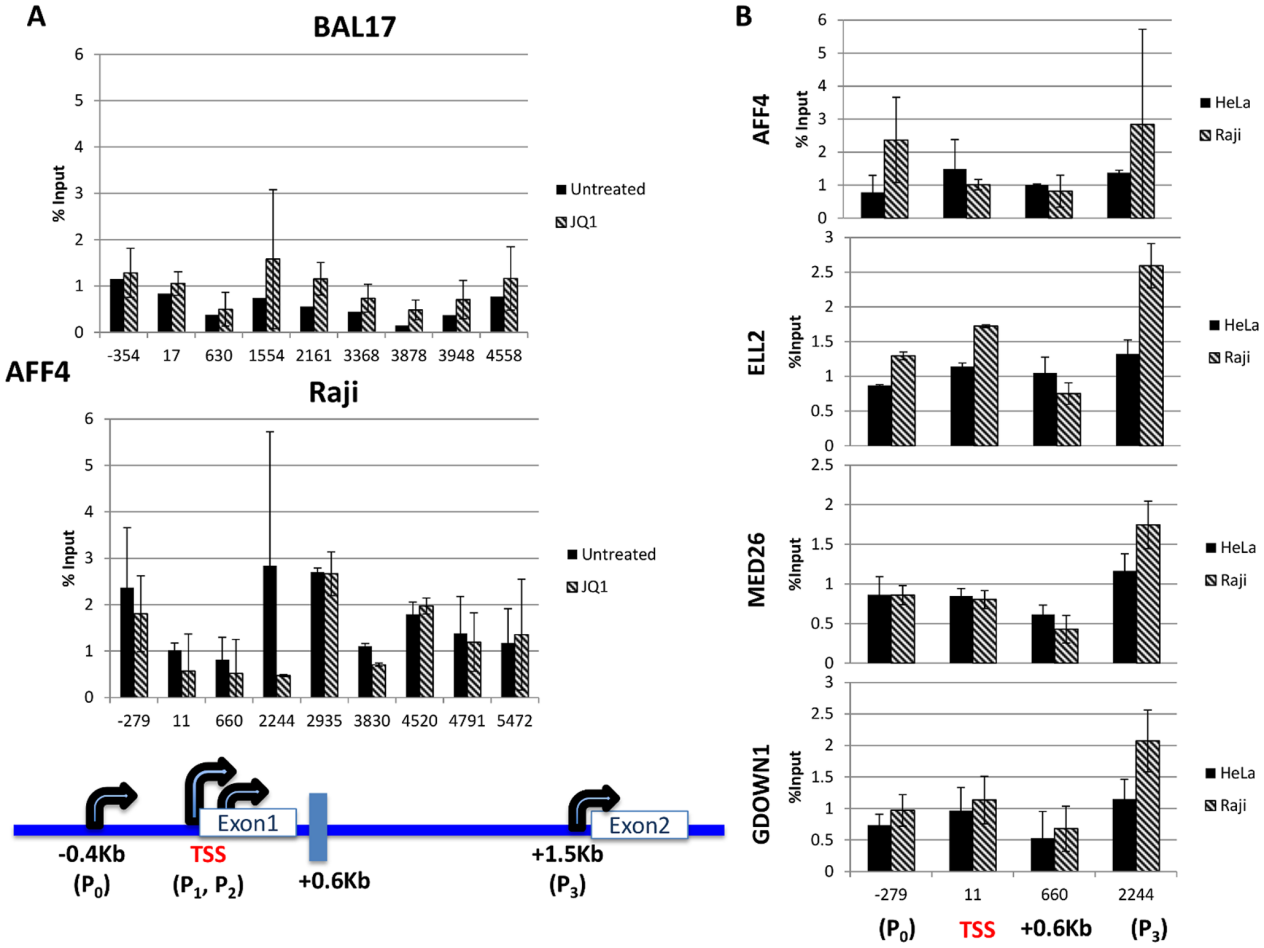
Occupancy of elongation factors at the *MYC* locus. Cells were either untreated or treated with 1 µM of JQ1 for 2 hours. (A) AFF4 was detected by ChIP across the length of the *MYC* gene in both BAL17 and Raji cells. (B) Detection of AFF4, ELL2 and MED26 in the promoter regions of untreated human HeLa and Raji cells. ChIP assays were performed in duplicate. Each experiment was analyzed in triplicate via real-time PCR, performed twice, and is reported as the mean and standard deviation of the two experiments.

P-TEFb frequently functions as a subunit of Super Elongation Complexes (SEC), consisting of P-TEFb and a mixture of one of three ELL family members, one of two EAF family members, one of two AF4 family members (AFF1 and AFF4), and either ENL or AF9 [29], [38]. As the SEC family of Pol II elongation factors are reported to contain the most catalytically active versions of P-TEFb active in high-level transcription and to regulate a checkpoint stage of transcription associated with elongation [29], [39], [40], we looked at SEC recruitment at *MYC* in different cell types. AFF4 is reported to serve as a central binding platform for elongation factors in many SEC formations and to target *MYC* and regulate its expression in cancer cells [39]. The level of AFF4 occupancy in BAL17 cells was low and refractory to JQ1 (Fig. 6A, top panel) but increased in the presence of JQ1. In contrast, AFF4 recruitment in Raji cells was much greater and sensitive to JQ1. Although the AFF4 promoter occupancy in Raji cells, particularly around 2244 site, was variable in the absence of JQ1, it was significantly decreased in the presence of JQ1. Moreover, consistent with the Pol II S2P and H3K36me3 results, JQ1 sensitivity was lost past the P_3_ promoter region (+2244), once again suggesting that the elongating transcription complex was refractory to JQ1 (Fig. 6A, bottom panel).

We subsequently looked at occupancy of another SEC component, ELL2, which has been reported to play a role in the transition from paused Pol II to elongation [40]. We also tested occupancy of the Mediator component (MED26) as well as Gdown1. Though first linked to initiation, a role for Mediator in recruiting Pol II transcription elongation factors and phosphorylation of the Pol II CTD regulating Pol II pausing and elongation has emerged [41]. The Mediator complex most strongly associated with Pol II includes MED26, which has been shown to increase activation of transcription in vitro to a greater degree than other forms of the Mediator complex and play a key role in SEC recruitment and MYC transcription [41]. Gdown1 is a Pol II binding protein shown to be necessary for a Mediator-dependent response to activation of transcription [42]. More appropriate to this study, Gdown1 has recently been shown to increase the stability of paused Pol II and regulate P-TEFb activity [28].

Because the available antibodies against ELL2, Med26 and Gdown1 were species (human) specific, we employed HeLa and Raji cells for these experiments. Given the differences focused around the P3 region, we focused on the general promoter region (P_0_, TSS, and P_3_) to test the differences between these cell lines. As observed for BAL17 cells, HeLa cells exhibited low AFF4 recruitment at this region in comparison to Raji cells (Fig. 6B). Consistent with AFF4 recruitment results, increased ELL2 occupancy near P_3_ was seen in Raji cells compared to HeLa cells. MED26 occupancy showed a pattern similar to that of other SEC components with an increase, though more modest, near the P_3_ promoter in Raji cells (Fig. 6B). Finally, we observed that Gdown1 followed a pattern similar to that of SEC and Mediator with an increase around the P_3_ promoter in Raji cells (Fig. 6B).

## Discussion

Due to the importance of *MYC* in the genesis of hematopoietic malignancies, intense efforts have been directed for the past two decades toward understanding its regulation. Yet, given that *MYC* overexpression is caused by a plethora of genetic lesions, a uniform set of rules governing its transcriptional regulation is still missing. Recent advances in this area include the discovery of a small molecule inhibitor, JQ1 that decreases *MYC* expression and is therapeutically effective in pre-clinical animal models of midline carcinoma and in BL cells. However, JQ1 does not inhibit *MYC* expression to a similar extent in all cancer cells, further underscoring that *MYC* transcription is perhaps under different controls in various cancer cell types. Consistent with this notion, we discovered that JQ1 inhibits *MYC* expression in all BL-derived cells tested but does not inhibit *MYC* expression in some other cancer cell lines, as observed earlier (19). Because *MYC* deregulation can occur via different modes, we hypothesized that in distinct lymphoma phenotypes, high-level *MYC* expression may come under the controls of different transcriptional and epigenetic regulatory mechanisms, which may be sensitive or resistant to JQ1/Brd4 inhibition. In this study, we explored these mechanisms to establish *MYC* transcriptional and epigenetic signatures associated with particular cell types with the hope that these signatures will better define lymphoma subtypes and provide new therapeutic avenues to explore for clinically aggressive B cell lymphomas.

Although BL cells are a heterogeneous lot with differences in the length of Ig/MYC translocation that reportedly translate to variable levels of *MYC* overexpression [43], BL cells in our hands (Fig. 1 and Figure S1, in File S1), and others [19], are uniformly susceptible to inhibition of *MYC* expression via Brd4 inhibition by JQ1. This inhibition appears to be regardless of whether these are EBV positive or negative BL lines (our study and ref 19). Brd4 is primarily associated with 5′-end promoters and enhancers [44], [45], [46], and while we do see a more prominent level of Brd4 at the TSS in Raji cells (Fig. 2), we also observe Brd4 occupancy across the body of the *MYC* gene in JQ1 resistant cells (Fig. 2). One function of Brd4 is P-TEFb recruitment and P-TEFb occupancy does parallel Brd4-inhibitor sensitive *MYC* transcription, despite the fact that there is little difference in P-TEFb occupancy in untreated cells of either resistant or sensitive cells. Therefore, it appears that in cells resistant to JQ1, a Brd4-independent mechanism operates to recruit or retain P-TEFb and produce high levels of *MYC* transcription.

**Figure 7:**
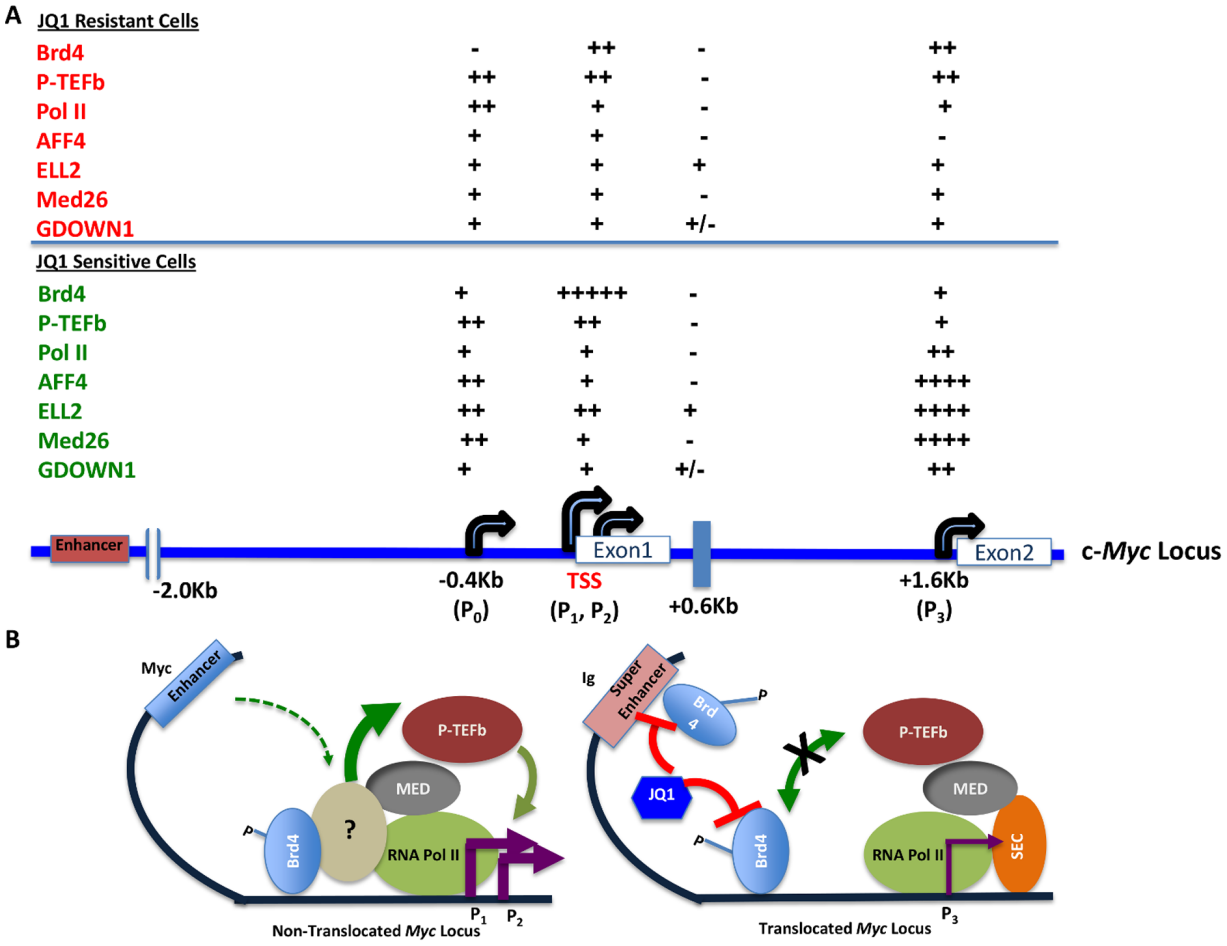
Proposed Model. (A) Summary of levels of factor occupancy spanning the *MYC* promoter P_0,_ P_1_, P_2_ and P_3_ regions and associated with regulation of *MYC* transcription. (B) Graphical representation of proposed model of *MYC* transcription elongation regulation under different conditions–left panel shows non-translocated *MYC* locus and right panel depicts *IGH-MYC* translocated locus.

Because the SEC components AFF4 and ELL2 play a role in *MYC* regulation and transcriptional elongation, we hypothesized that an increase in these co-factors might relax Brd4 requirements. Surprisingly, their increased presence actually correlated with increased requirements for Brd4 (Fig. 6). The position of increased SEC components together with MED26 and Gdown1 around the P3 promoter suggests high transcriptional activity or a regulatory checkpoint in this region that is not present in cells that are resistant to JQ1 (summarized in Figure 7). It has been noted that in BL cells, due to *MYC-IG* translocation, the otherwise minor P3 promoter often becomes active and correlates with higher MYC expression [43]. Coupled with the recent demonstration that the translocated *MYC* locus harbors super enhancers that are sensitive to JQ1 [45], this raises the possibility that the mechanism of transcriptional regulation of *MYC* involving its native/non-translocated enhancer is different (Fig. 7B, Figure S2, in File S1).

Regulation of transcription at the *MYC* minor promoters, including P3, has been shown to involve the P-TEFb regulator 7SK snRNA, which participates in transformation-dependent *MYC* deregulation [47]. 7SK snRNA is a complex of RNA and proteins such as LARP7 and HEXIM1 that are known to stabilize P-TEFb in an inactive form [13], [14], [47]. JQ1 also leads to the release of P-TEFb from the 7SK snRNP and to HIV gene expression that involves an increase in AFF4 and ELL2 [48], [49]. Moreover, while JQ1 inhibited *MYC* expression in BL lines tested, it enhanced BCR stimulated induced transcription of c-*fos* in these cells (Figure S1, in File S1). Thus, the effects of Brd4 inhibition by JQ1 appear more complicated than has been previously anticipated.

Much is known about the different *MYC* promoters and their possible functions in oncogenesis. BAL17 and HeLa cells may behave like GLC4 cells, where *MYC* is transcribed at a high rate but not grossly rearranged [50]. That *MYC* transcription in GLC4 is reported to initiate mainly from P1 instead of the P2 promoter and that this promoter shift seems to overcome transcript termination at a transcriptional pause site described for Burkitt’s lymphoma [50]. In the future, it will be interesting to conclusively determine if any difference in cell-specific primary transcription and *MYC* promoter usage is driving, or being driven by, the differences in transcriptional elongation regulation we report here. Point mutations could also play a role in differential JQ1 sensitivity though a search of available UCSC Genome Browser tracks only finds one SNP reported in the general vicinity of the P1/P2 promoter (data not shown).

Altered regulation at the elongation stage of transcription has been proposed to contribute to leukemic pathogenesis [29], [51]. While many PRGs such as *MYC* have a well-recognized checkpoint at +40 to the TSS that is associated with paused transcription [52], [53], our data indicate that an additional potential checkpoint near the region around P3 may be utilized differently depending upon the mode of *MYC* overexpression. In the near future, we hope to employ different *MYC*-driven lymphoma samples to test whether the transcriptional signatures that we observe here correlate with the particular *MYC* genetic lesion (translocated versus non-translocated).

## Supporting Information

File S1 **File includes Figures S1–S3.** Figure S1. Effect of JQ1 on *MYC* and *Fos* expression. (A) Effect of JQ1 on Ramos BL cell line in the absence of any stimulation. (B) The reported cell lines were incubated at 37^C^ with 1 µM JQ1 for 2 hours prior to addition of 10 µM of anti-mouse IgM fragments, which triggers the B cell receptor (BCR). After 30 minutes stimulation, RNA was harvested and analyzed for c-*fos*, c-*myc* and ActB mRNA expression as detailed in Materials and Methods. The experiments were performed in triplicate and reported as the mean and standard deviation of the ratio of target mRNA over ActB mRNA. Figure S2. RNA Pol II occupancy at the MYC locus in Raji and HeLa cells. ChIP-seq data–ENCONDE UCSC Genome Browser tracks of Polymerase II occupancy at the *MYC* locus in HeLa and Raji cells. Figure S3. Primer positions and sequences used for ChIP across mouse and human *MYC* locus.
(PDF)
