# Correction: Regulation of *MYC* Expression and Differential JQ1 Sensitivity in Cancer Cells

**DOI:** 10.1371/journal.pone.0126328

**Published:** 2015-04-22

**Authors:** Trent Fowler, Payel Ghatak, David H. Price, Ronald Conaway, Joan Conaway, Cheng-Ming Chiang, James E. Bradner, Ali Shilatifard, Ananda L. Roy

There is an error in Fig 2 that appears to have occurred during the preparation of files after the manuscript was accepted. In Fig 2B, the blot for Brd4 incorrectly appears as a duplicate of P-Brd4. The authors have provided a corrected version of Fig 2 here.

**Fig 2:**
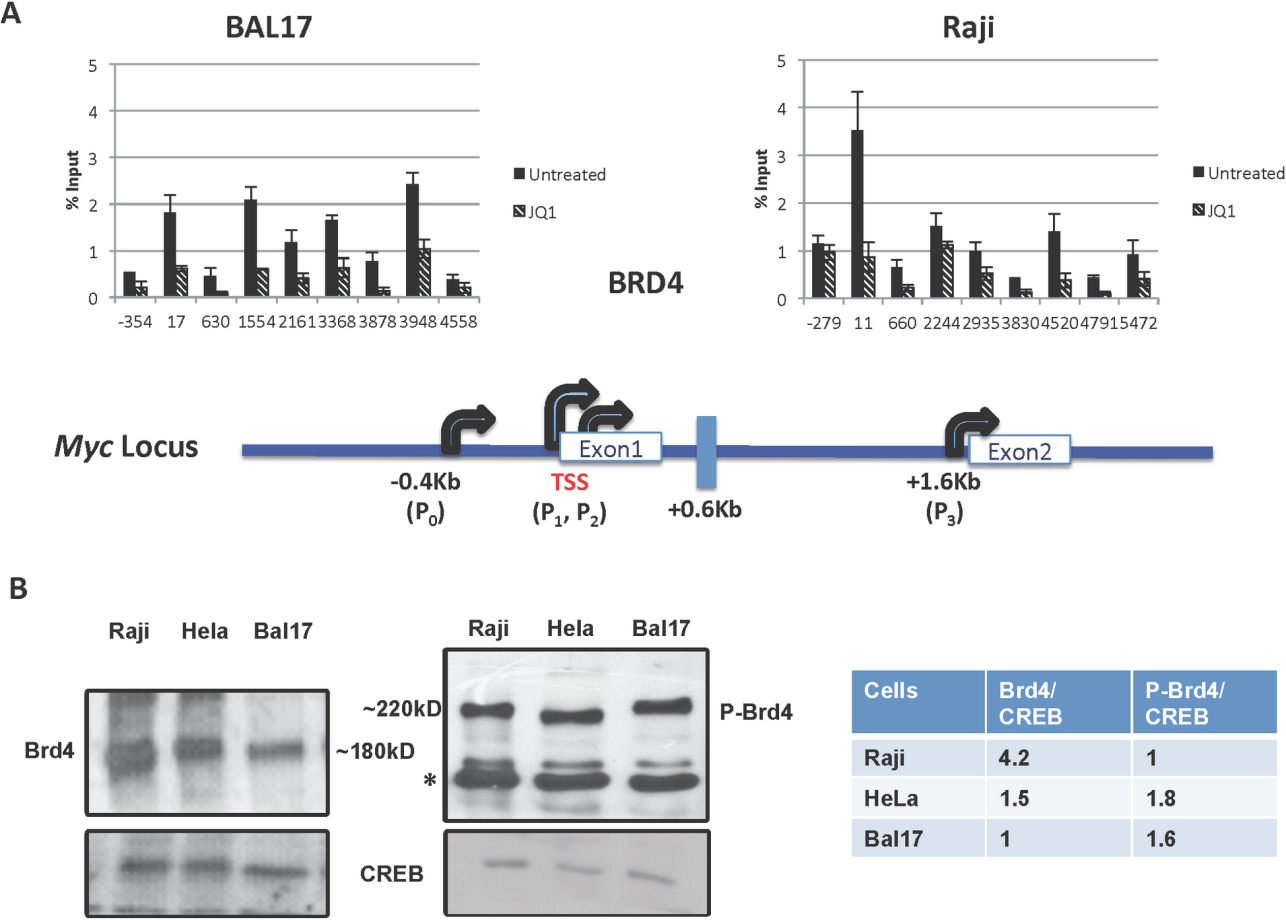
Brd4 occupancy and expression in different cells. Cells were either untreated or treated with 1 μM of JQ1 for 2 hours. (A) Chromatin Immunoprecipitation (ChIP) across *MYC* with anti-C-terminal Brd4 antibody. Each experiment was performed twice, analyzed in triplicate via real-time PCR and reported as the mean and standard deviation of the two experiments. A representation of the promoter area of *MYC* is provided for orientation. (B) Western blotting to detect (far left) Brd4 (~180 KD) and (middle) Brd4-S484/488-phos (P-Brd4, ~220 KD) was performed three times. A non-specific band detected with phopsho-Brd4 antibody is denoted with an asterisk. Typical results are shown with densitometry analysis relative to CREB expression, which is used as a normalization control (far right).
