# Supplementary figures and images for: Regulation of MYC Expression and Differential JQ1 Sensitivity in Cancer Cells
Source: PLoS One. 2014 Jan 23;9(1):e87003. doi: 10.1371/journal.pone.0087003 (PMC3900694; doi:10.1371/journal.pone.0087003)

Figure S1

**A**

**Ramos**

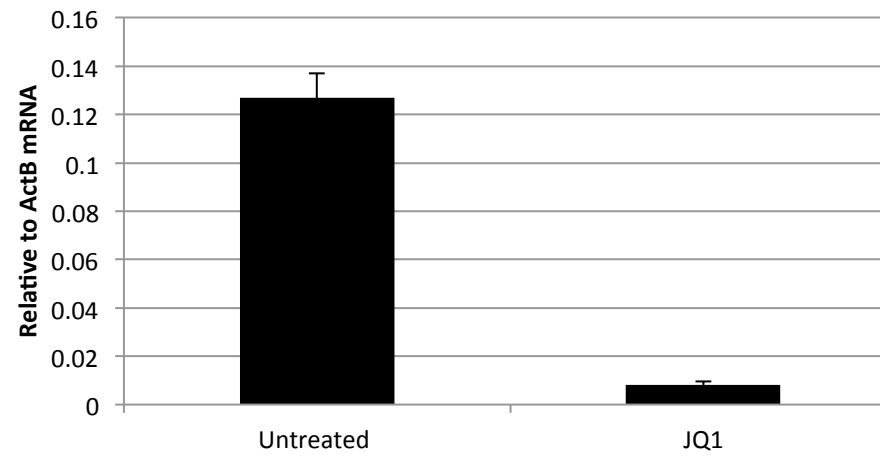

**B**

**BAL17**

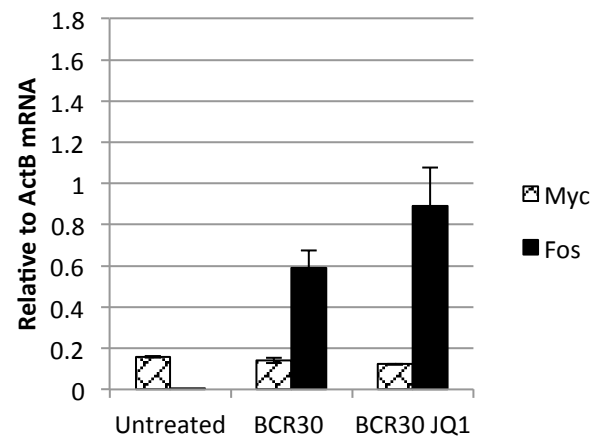

**Akata**

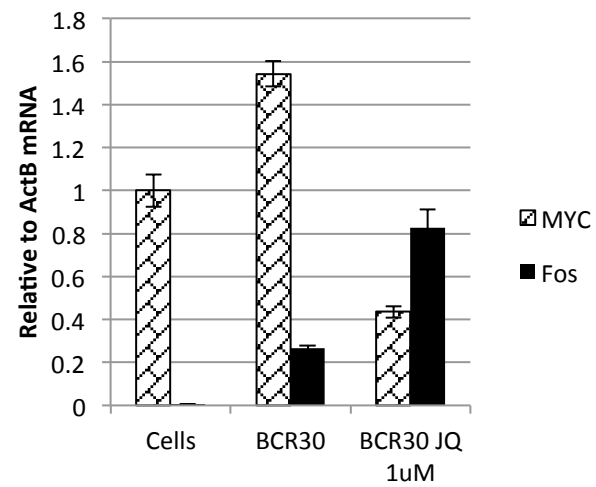

**Ramos**

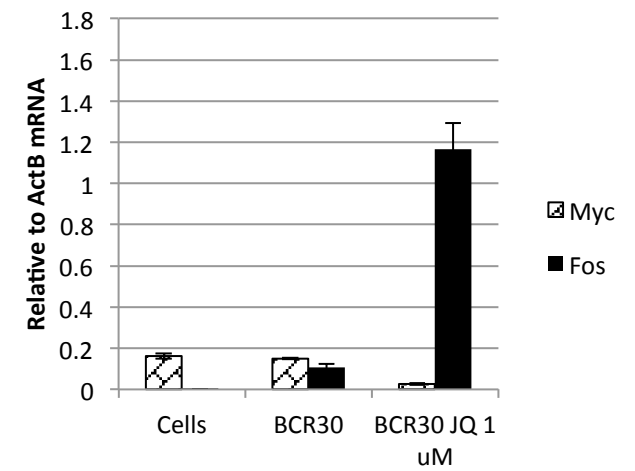

Figure S2

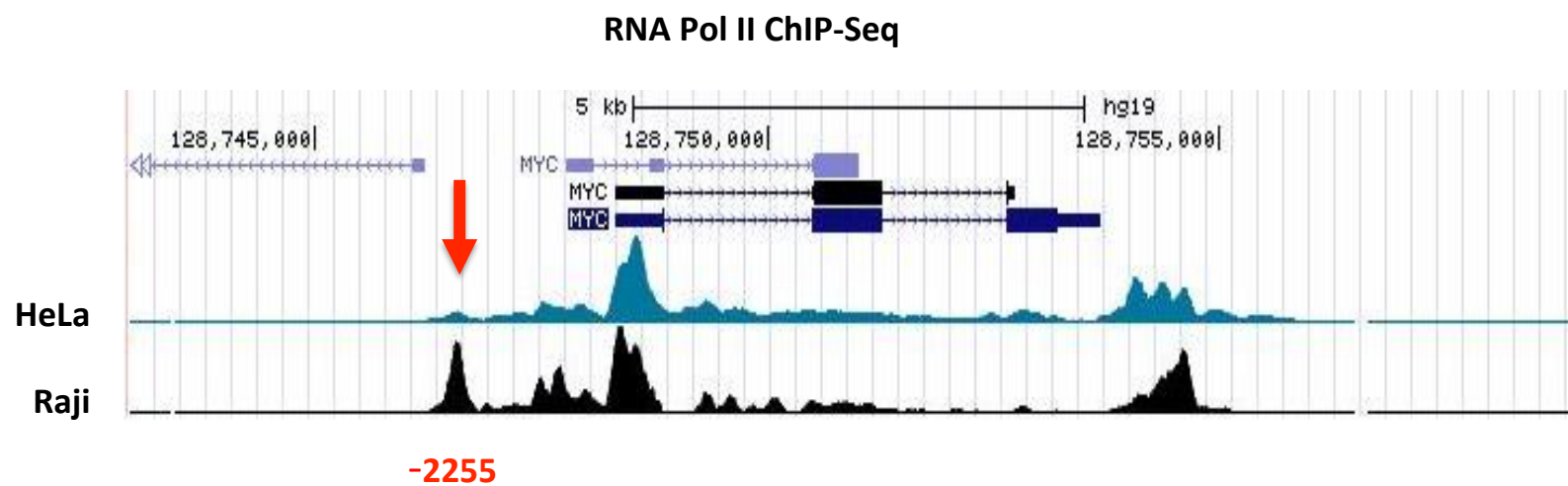

# MYC PCR PRIMERS

Figure S3

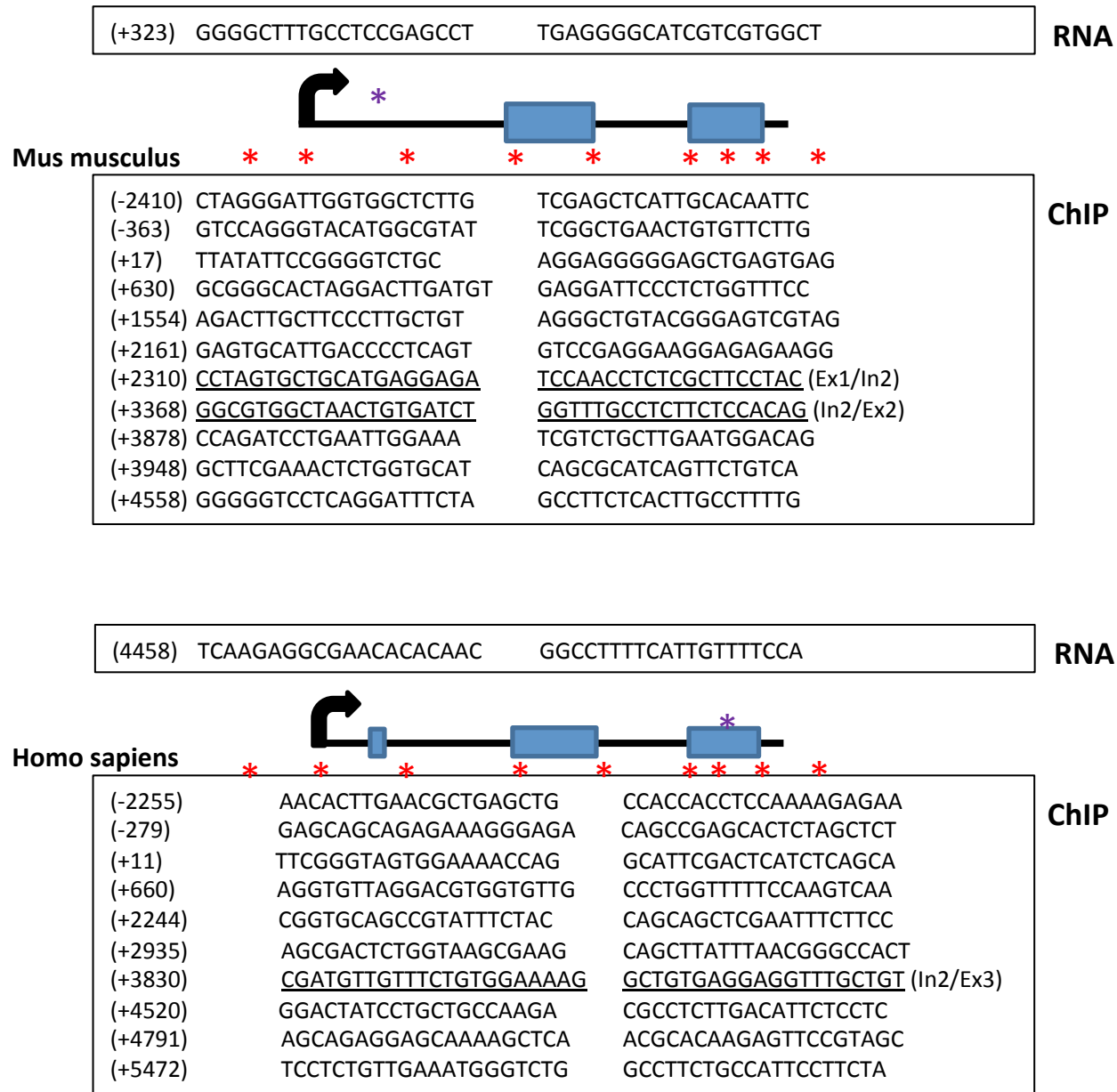

Supplement: File S1 — File includes Figures S1–S3. Figure S1. Effect of JQ1 on MYC and Fos expression. (A) Effect of JQ1 on Ramos BL cell line in the absence of any stimulation. (B) The reported cell lines were incubated at 37C with 1 µM JQ1 for 2 hours prior to addition of 10 µM of anti-mouse IgM fragments, which triggers the B cell receptor (BCR). After 30 minutes stimulation, RNA was harvested and analyzed for c-fos, c-myc and ActB mRNA expression as detailed in Materials and Methods. The experiments were performed in triplicate and reported as the mean and standard deviation of the ratio of target mRNA over ActB mRNA. Figure S2. RNA Pol II occupancy at the MYC locus in Raji and HeLa cells. ChIP-seq data–ENCONDE UCSC Genome Browser tracks of Polymerase II occupancy at the MYC locus in HeLa and Raji cells. Figure S3. Primer positions and sequences used for ChIP across mouse and human MYC locus. (PDF) [file pone.0087003.s001.pdf]
